# Uma Luta Passo a Passo pela Vida em uma Jovem com Embolismo Pulmonar de Alto Risco e Oclusão Bilateral da Artéria Renal

**DOI:** 10.36660/abc.20210189

**Published:** 2022-02-14

**Authors:** Miruna Stefan, Roxana Cristina Rimbas, Rozina Vornicu, Ruxandra Daneţ, Vlad Damian Vintila, Bogdan Dorobat, Alexandra Carp, Vinereanu Dragos

**Affiliations:** 1 University Emergency Hospital Bucharest Bucharest Romênia University Emergency Hospital Bucharest – Cardiology, Bucharest – Romênia; 2 Carol Davila University of Medicine and Pharmacy Bucharest Romênia Carol Davila University of Medicine and Pharmacy – Cardiology, Bucharest – Romênia

**Keywords:** Embolia Pulmonar, Imagem Multimodal, Terapia Trombolítica

## Introdução

Os trombos em trânsito no átrio direito são raros no contexto da embolia pulmonar aguda (EPA) e estão associados a alta mortalidade e morbidade.^1 , 2^ Deve-se sempre realizar um ecocardiograma transtorácico (ETT) em pacientes que apresentam dispneia, dor torácica e instabilidade hemodinâmica. Este caso ilustra a importância da avaliação multimodal por imagem no tratamento de casos difíceis com EPA e trombo em trânsito no átrio direito, bem como das complicações dessa doença. A abordagem terapêutica deve ser decidida por equipes multidisciplinares.

### Apresentação do caso

Uma paciente do sexo feminino, obesa, de 48 anos, com hipertensão arterial e diabetes mellitus tipo 2 controlados, foi transferida para uma coronariografia em nossa unidade de terapia intensiva (UTI), com suspeita de infarto do miocárdio com supradesnivelamento do segmento ST (STEMI) em derivações do ventrículo direito e inferior e choque cardiogênico. Ela havia comparecido a outro departamento de emergência (DE) com dor torácica constritiva e dispneia grave que havia começado 12 horas antes. Ela havia sido diagnosticada com anemia microcítica hipocrômica moderada um ano antes, sem maiores investigações. O exame clínico no DE identificou pele pálida, fria e com sudorese. A pressão arterial (PA) era 80/60 mmHg. Ela apresentava pulso fraco e índice de respiração de 40/min. O hemograma revelou anemia microcítica hipocrômica moderada (Hb 8,2 g/dl), hiperglicemia (309 g/dl), citólise hepática leve (AST 119 U/L, ALT 75 U/L), aumento da quinase da creatinina e da quinase-MB da creatinina (CK 552 U/L, CKMB 55 U/L), acidose metabólica com compensação lática (pH 7,41, ácido lático 5,6 mmol/L, BE-11,4mmol/L, pO2 151mmHg, pCO2 20,9mmHg). A troponina I de alta sensibilidade (hs-cTnI) era de 3558 ng/L e o peptídeo natriurético cerebral N-terminal (NTproBNP) era 7380 pg/ml. O teste RTPCR SARSCOV2 foi negativo. Não havia sinais de insuficiência renal. O eletrocardiograma demonstrou taquicardia sinusal, bloqueio do ramo do feixe direito incompleto intermitente (BRFD), padrão S1Q3T3, supradesnivelamento do segmento ST em D3, aVF, V1-V2 e V3R-V5R, e onda T negativa nas derivações V1-V3 (Material suplementar 1). O raio X do tórax revelou cardiomegalia e artérias pulmonares aumentadas (Material suplementar 2). Ela recebeu doses de ataque de aspirina e clopidogrel e foi encaminhada a nosso hospital. Ao ser admitida em nosso DE, a paciente estava em choque cardiogênico, com suporte de vasopressores e inotrópicos. Ela também apresentava metrorragia leve. Não havia sinais de trombose venosa profunda.

**Figura 1:**
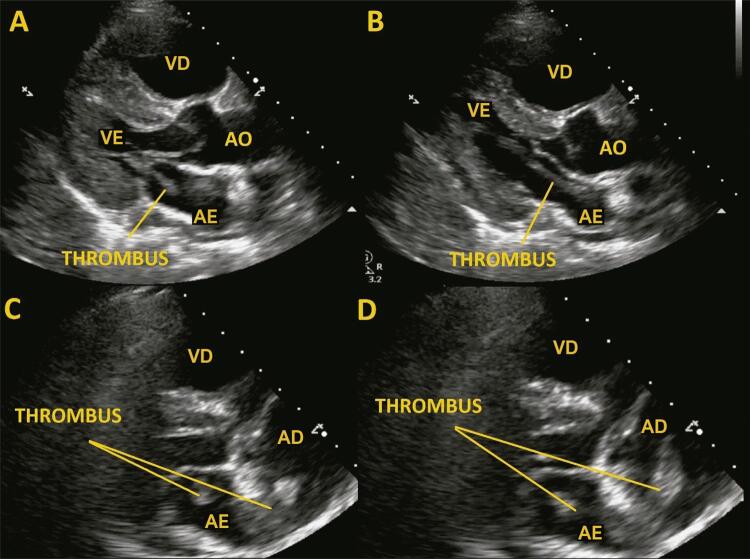
Ecocardiograma transtorácico. Painéis A, B – vista do eixo longo paraesternal mostrando trombo grande serpiginoso estendendo-se do átrio esquerdo, através da válvula mitral, ao ventrículo esquerdo. Painéis C, D – vista modificada de 4 câmaras mostrando as câmaras cardíacas direitas aumentadas e o trombo móvel que se estende do átrio direito ao átrio esquerdo, cruzando uma comunicação interatrial, através da válvula mitral. AO: aorta; AE: átrio esquerdo; AD: átrio direito: VE: ventrículo esquerdo; VD: ventrículo direito.

**Figura 2:**
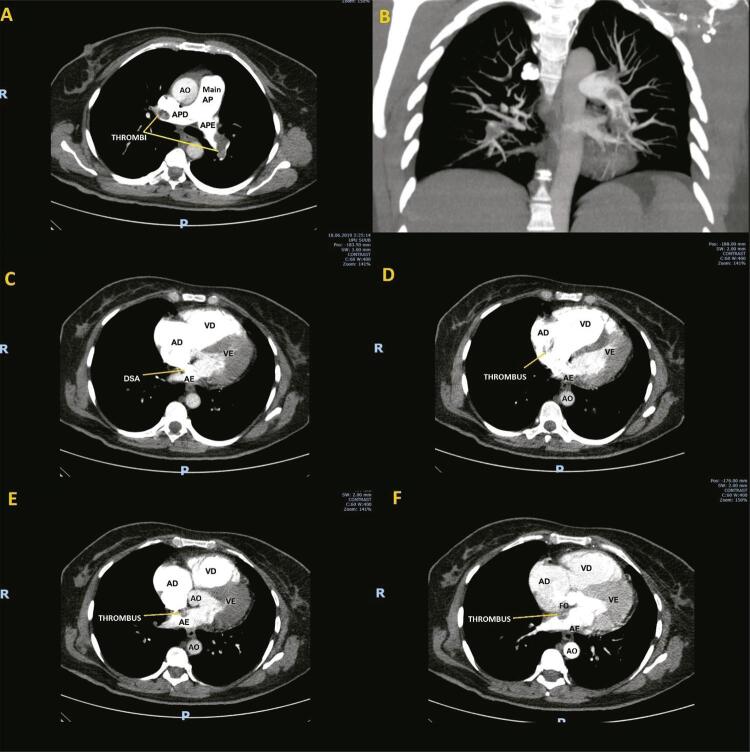
Tomografia computadorizada torácica. Painel A - seção transversal mostrando grande embolia pulmonar com trombo nas principais artérias pulmonares esquerda e direita. Painel B - Imagem de reconstrução mostrando a oclusão das principais artérias pulmonares e oligoemia periférica. Painel C - seção transversal mostrando o defeito de septo atrial com o contraste fluindo do átrio direito aos átrios esquerdos. Painel D - seção transversal mostrando o trombo no átrio direito. Painel E - seção transversal mostrando o trombo se estendendo para o átrio esquerdo. Painel F - seção transversal mostrando o trombo na fossa oval. AP: artéria pulmonar; APD: artéria pulmonar direita; APE: artéria pulmonar esquerda; AO: aorta; VD: ventrículo direito; AD: átrio direito; VE: ventrículo esquerdo; AE: átrio esquerdo; DSA: defeito de septo atrial; FO: fossa oval.

O ETT de emergência foi realizado, de acordo com nossas diretrizes internas, antes de a paciente ser encaminhada para o laboratório de cateterização. Surpreendentemente, este procedimento apresentou câmaras cardíacas direitas extremamente dilatadas, com regurgitação tricúspide grave, septo interventricular achatado, acinesia da parede livre do ventrículo direito (VD) com contratilidade apical preservada (sinal de McConnell) e disfunção grave do VD (TAPSE = 14 mm), hipertensão pulmonar, com sinais de débito cardíaco baixo. Havia um trombo móvel serpiginoso muito longo que se estendia do átrio direito (AD) ao átrio esquerdo (AE) em uma comunicação interatrial, através das válvulas mitral e aórtica ( Figura 1 ; Vídeo 1; Material suplementar 3, 4). Foi iniciada a infusão contínua de heparina não fracionada e o suporte vasoativo foi continuado. A tomografia computadorizada revelou embolia pulmonar grande com um trombo pulmonar em sela que se estendia a todos os ramos das artérias pulmonares esquerda e direita, ocluindo as artérias quase completamente. A TC também confirmou a presença de uma massa intracardíaca heterogênea densa que se estendia do AD ao AE por um defeito de septo interatrial ( Figura 2 ). Nesse ponto, foi confirmada a EP de alto risco com trombo intracardíaco extremamente móvel.

Comprovou-se que o diagnóstico de STEMI inferior estava incorreto e, consequentemente, não foi realizada a coronariografia.

A paciente tinha um índice de gravidade da embolia pulmonar simplificado (sPESI) de 2, que indicava um risco de mortalidade de 30 dias de 10,9% [1]. Por outro lado, a paciente tinha alto risco de sangramento (anemia moderada, metrorragia leve contínua, e doses de ataque de aspirina e clopidogrel). Embora a trombólise farmacológica representasse um risco duplo de embolização sistêmica e sangramento, a paciente apresentava sinais graves de baixo débito cardíaco e disfunção grave do VD. Optou-se pela trombólise sistêmica com alteplase com infusão contínua de heparina não fracionada, considerando que não havia possibilidade de embolectomia no local e a condição do paciente era potencialmente fatal.

**Figura 3:**
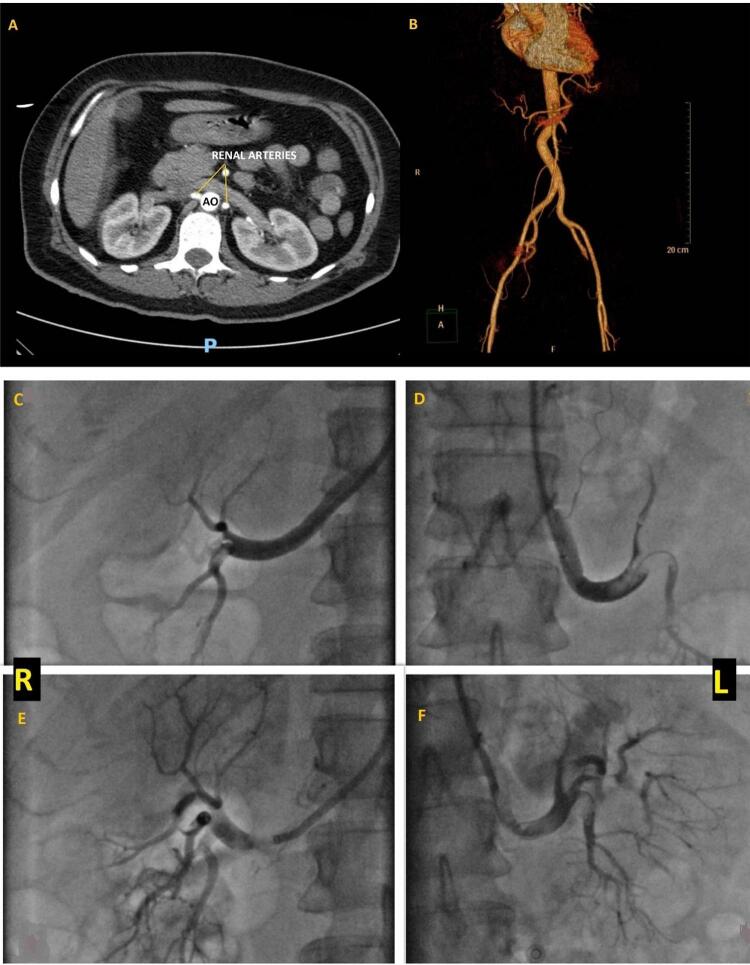
Tomografia computadorizada abdominal após a trombólise, mostrando a oclusão de ambas as artérias renais. Painel A - seção transversal - artérias renais são vistas apenas na emergência, seguidas de oclusão completa de ambas as artérias. Painel B - reconstrução 3D mostrando ausência de fluxo sanguíneo nas artérias renais. Painéis C e D: ausência de fluxo/fluxo baixo nas artérias renais direita e esquerda, antes da trombectomia. Painéis E e F: fluxo em ambas as artérias renais após a trombectomia por aspiração bem-sucedida.

Houve uma melhoria gradual inicial dos parâmetros hemodinâmicos após a trombólise. A PA aumentou para 100/60mmHg, com dose baixa de suporte vasopressor, e aumento da saturação de oxigênio para 98%. Uma nova ETT não detectou trombos intracardíacos (Material suplementar 5). Uma hora após o final da trombólise, a paciente apresentou dor abdominal intensa, anúria e hematúria, associadas a uma queda dos níveis de hemoglobina de 2 g/dl. Uma TC abdominal de emergência demonstrou oclusão completa de ambas as artérias renais ( Figura 3 , Painéis A e B), sem sinais de complicações hemorrágicas. Uma equipe multidisciplinar, composta de cardiologistas, radiologistas e intensivistas, optou pela intervenção por trombectomia por aspiração, para salvar a função renal, apesar do alto risco de hemorragia, com uma clara melhoria do fluxo renal comprovada por imagens, porém sem melhorias clínicas e biológicas ( Figura 3 , Painéis C, D, E e F).

Depois do procedimento intervencionista, ela foi transferida para a unidade de terapia intensiva para receber suporte avançado e diálise. A heparina não fracionada foi continuada. Entretanto, a paciente desenvolveu grave falência múltipla de órgãos e morreu 24 horas após a internação.

## Discussão

A EPA de alto risco, com grande trombo hipermóvel preso em uma comunicação interatrial é muito rara e envolve alta mortalidade e morbidade, incluindo uma probabilidade aumentada de embolia paradoxal.^2^

Neste caso, havia um trombo “em-trânsito”^3^ que se estendia do AD, tanto através de uma comunicação interatrial com o AE e através da válvula mitral para o ventrículo esquerdo quanto através da válvula tricúspide para o ventrículo direito.

O tratamento ideal desse cenário clínico é muito debatido. As estratégias de tratamento incluem trombólise, anticoagulação, e/ou extração cirúrgica, sem consenso sobre a superioridade de qualquer uma delas.^4^ A terapia trombolítica sistêmica é a atitude recomendada para a EP de alto risco (classe I, nível B).^1^ Como alternativa, a embolectomia pulmonar cirúrgica (classe I, nível C) deve ser considerada para pacientes com EP de alto risco, para os quais a trombólise é contraindicada.^1^ A experiência recente corrobora a combinação de oxigenação por membrana extracorpórea (ECMO) e embolectomia cirúrgica, em pacientes com EP de alto risco com ou sem ressuscitação cardiopulmonar.^1^ A embolectomia cirúrgica demonstrou uma tendência para melhorar a sobrevida, mas o índice de mortalidade no pós-operatório é alto.^5^ Ensaios randomizados em relação às opções de tratamento (cirúrgico ou médico) não são viáveis devido à raridade da condição, e a literatura se baseia em relatos de casos.

A trombólise aumenta o risco de fragmentação do trombo e embolização distal. Um relato feito por Chartier et al. mostrou que não havia diferença significativa entre anticoagulação com heparina, trombólise ou remoção cirúrgica em termos de mortalidade durante a internação.^6^ Torbicki et al.^4^ afirmam que o resultado favorável após a trombólise pode estar relacionado à maior disponibilidade e o curto período entre o diagnóstico e o tratamento. Ferrari et al.^7^ demonstraram que, após a trombólise, 50% dos coágulos desapareceram em até 2 horas, e os demais entre 12 e 24 horas. Alguns estudos afirmam que, no caso de EPA de alto risco com trombo intracardíaco, a melhor atitude terapêutica é a trombectomia cirúrgica, especialmente no caso de pacientes com comunicação interatrial.^8^ Entretanto, a trombectomia cirúrgica, além de não estar prontamente disponível em muitos centros, traz o risco de atraso no tratamento, anestesia geral e by-pass cardiopulmonar. Em uma análise sistêmica, a trombectomia cirúrgica foi associada a uma mortalidade de 30 dias de 10,8%, significativamente mais baixa se comparada à trombólise (26,3%) e à anticoagulação (25,6%).^9^

As diretrizes recomendam o uso de trombolíticos direcionados por cateter em EP de risco intermediário a alto e o uso de trombectomia direcionada por cateter em pacientes com contraindicações absolutas a trombolíticos ou terapia trombolítica fracassada. Entre as abordagens percutâneas que foram usadas separadamente ou em combinações em pacientes com contraindicação absoluta à trombólise estão: trombectomia por aspiração e fragmentação do trombo, trombectomia reolítica, embolectomia por sucção, e trombólise assistida por ecocardiograma.^10^

Entretanto, as técnicas de fragmentação do trombo implicam em um risco significativo de embolização distal e proximal. Os cateteres de trombectomia reolítica funcionam criando um vácuo por trás de uma área de jatos de solução salina de alta pressão na ponta do cateter, que permite a fragmentação e a aspiração do trombo simultaneamente. A embolectomia por sucção é capaz de realizar a retirada do trombo sem os efeitos colaterais associados às técnicas de fragmentação e reolíticas.^10^ A trombólise direcionada por cateter com auxílio de ultrassom, que combina a emissão de ultrassom de baixa energia e infusão de um agente trombolítico por um cateter contendo vários furos laterais, é superior à anticoagulação apenas por heparina para melhorar a dilatação do ventrículo direito no período de 24 horas sem grandes complicações de sangramento, mas continua estando raramente disponível.^10^

Conforme sugerido em diretrizes atuais, essas estratégias de tratamento poderiam ter sido de grande valor neste caso. Ainda assim, não há estudos de comparação direta entre os vários sistemas ou comparando terapias direcionadas por cateter a trombólise sistêmica. Portanto, a escolha de modalidade deve ser baseada em experiência local e disponibilidade. Nenhuma das estratégias de tratamento mencionadas acima estava disponível em nosso hospital e a paciente estava instável demais para ser transportada a outros hospitais. Além disso, o shunt cardíaco direita-esquerda e a trombose esquerda concomitante, como era este caso, são contraindicações absolutas da trombólise direcionada por cateter.^10^

Neste caso, optou-se pela trombólise sistêmica que estava imediatamente disponível, porque o paciente tinha disfunção grave no VD e débito cardíaco baixo grave.

Mais recentemente, no contexto da pandemia da Covid-19, houve relatos de casos de EPA e trombo em trânsito através de forame oval patente, 28 dias após o aparecimento de sintomas de Covid.^10^ O estado pró-inflamatório e pró-trombótico explica a hipercoagulabilidade associada à infecção por SARS-CoV-2.^11^ Ainda não se sabe bem quanto tempo esse estado de hipercoagulabilidade persiste após a recuperação da infecção.^11^ São necessários mais dados para determinar a duração ideal da anticoagulação.^11^ Não há dados relacionados à trombectomia cirúrgica nesse contexto.

## Conclusões

Apresenta-se aqui um caso muito raro de embolia pulmonar de alto risco, com um grande trombo hipermóvel preso em um defeito de septo interatrial e que cruza as válvulas aórtica e mitral, trazendo também um alto risco de embolia sistêmica. A paciente passou por uma complicação embólica fatal ainda mais rara após a trombólise sistêmica, a oclusão bilateral da artéria renal. Este caso ilustra as decisões difíceis que os clínicos podem vir a enfrentar em sua prática diária. Foi necessário avaliar os prós e os contras da trombólise farmacológica, uma vez que a embolectomia cirúrgica não era uma possibilidade, e as evidências da superioridade da embolectomia cirúrgica em pacientes hemodinamicamente instáveis com disfunção grave do VD ainda são controversas. A utilidade da avaliação multimodal por imagem em todas as etapas de decisão de tratamento também é enfatizada.

## * Material suplementar

Para verificar as figuras, por favor, clique aqui.

Para assistir ao Vídeo 1, por favor, clique aqui.

Para assistir ao Material Suplementar 3, por favor, clique aqui.

Para assistir ao Material Suplementar 4, por favor, clique aqui.

Para assistir ao Material Suplementar 5, por favor, clique aqui.
