# Supplementary material for: Uma Luta Passo a Passo pela Vida em uma Jovem com Embolismo Pulmonar de Alto Risco e Oclusão Bilateral da Artéria Renal
Source: Arq Bras Cardiol. 2022 Feb 14;118(2):530–5. [Article in Portuguese] doi: 10.36660/abc.20210189 (PMC8856696; doi:10.36660/abc.20210189)
Supplement: Translation

# Translation: A Step-By-Step Fight for Life in a Young Woman with High-Risk Pulmonary Embolism and Bilateral Renal Artery Occlusion

**Authors:** Miruna Stefan, Roxana Cristina Rimbas, Rozina Vornicu, Ruxandra Daneţ, Vlad Damian Vintila, Bogdan Dorobaţ, Alexandra Carp, Vinereanu Dragos

**Affiliations:** 1 Romania University Emergency Hospital Bucharest – Cardiology, Bucharest – Romania; 2 Romania Carol Davila University of Medicine and Pharmacy – Cardiology, Bucharest – Romania

**Keywords:** Pulmonary Embolism, Multimodal Imaging, Thrombolytic Therapy

## Introduction

Right heart thrombus-in-transit is rare in the context of acute pulmonary embolism (APE) and is associated with high mortality and morbidity.^1 , 2^ Transthoracic echocardiography (TTE) should always be performed in patients presenting dyspnea, chest pain, and hemodynamic instability. This case illustrates the importance of multimodality imaging in managing difficult cases with APE and right heart thrombus in transit, as well as the complications of this condition. A therapeutic approach should be decided on by multidisciplinary teams.

### Case presentation

A 48-year-old obese woman, with controlled arterial hypertension and type 2 diabetes mellitus was transferred for coronarography to our cardiac care unit (CCU) with presumed ST-elevation myocardial infarction (STEMI) in the inferior and right ventricle leads, coupled with cardiogenic shock. She was admitted to another emergency department (ED) with constrictive chest pain and severe dyspnea that had started 12h earlier. She was diagnosed with moderate hypochromic microcytic anemia one year before, without further investigations. Clinical exam in ED revealed pale, cold, sweating skin. Blood pressure (BP) was 80/60mmHg. She had a weak pulse and a respiration rate of 40/min. Blood tests showed moderate hypochromic microcytic anemia (Hb 8.2g/dl), hyperglycemia (309mg/dl), mild hepatic cytolysis (AST 119 U/L, ALT 75 U/L), increased creatine kinase and creatine kinase-MB (CK 552 U/L, CKMB 55 U/L), and metabolic acidosis with respiratory compensation (pH 7.41, lactic acid 5.6 mmol/L, BE-11.4mmol/L, pO2 151mmHg, pCO2 20.9mmHg). High-sensitivity Troponin I (hs-cTnI) was 3,558 ng/L and N-terminal-pro-brain-natriuretic-peptide (NTproBNP) was 7,380 pg/ml. RTPCR SARSCOV2 was negative. There were no signs of renal impairment. The electrocardiogram showed sinus tachycardia; intermittent incomplete right bundle branch block (RBBB); S1Q3T3 pattern; ST-segment elevation in D3; aVF, V1-V2, and V3R-V5R; and a negative T wave in V1-V3 leads (Supplementary material 1). The chest X-ray revealed cardiomegaly and enlarged pulmonary arteries (Supplementary material 2). The patient received loading doses of aspirin and clopidogrel and was sent to our hospital. On admission to our ED, the patient was in cardiogenic shock, on vasopressor and inotrope support. She also had mild metrorrhagia. There were no signs of deep venous thrombosis.

Emergency TTE was performed, according to our internal guidelines, before sending the patient to the catheterization laboratory. Surprisingly, this procedure showed extremely dilated right heart chambers, with severe tricuspid regurgitation, a flattened interventricular septum, right ventricle (RV) free wall akinesia with preserved apical contractility (McConnell’s sign), and severe RV dysfunction (TAPSE=14mm), pulmonary hypertension, with signs of low cardiac output. There was a very long serpiginous, mobile thrombus extending from the right atrium (RA) to the left atrium (LA) across an interatrial communication, through the mitral and aortic valves ( Figure 1 ; Video 1; Supplementary material 3, 4). A continuous infusion of unfractionated heparin and continued vasoactive support were implemented. A CT scan showed a massive pulmonary embolism with a saddle pulmonary thrombus extending into all the branches of the left and right pulmonary arteries, occluding the arteries almost completely, and confirmed the presence of a heterogenous, dense intracardiac mass extending from the RA to the LA through an interatrial septal defect ( Figure 2 ). At this point, high-risk PE with intracardiac extremely mobile thrombus was confirmed.

**Figure 1:** Transthoracic echocardiography. Panel A, B – parasternal long axis view showing large, serpiginous thrombus extending from the left atrium, through the mitral valve, into the left ventricle. Panel C, D – modified 4-chamber view showing enlarged right heart chambers and mobile thrombus extending from the right atrium to the left atrium, across an interatrial communication, through the mitral valve. AO: aorta; LA: left atrium; RA: right atrium; LV: left ventricle; RV: right ventricle.

**Figure 2:** Thoracic computed tomography. Panel A - transversal section showing massive pulmonary embolism with thrombus in the main left and right pulmonary arteries. Panel B - Reconstruction image showing occlusion of the main pulmonary arteries and peripheral oligoemia. Panel C - transversal section showing the atrial septal defect with the contrast substance flowing from the right atrium to the left atriums. Panel D - transversal section showing the thrombus in the right atrium. Panel E - transversal section showing the thrombus extending to the left atrium. Panel F - transversal section showing the thrombus in the fosa ovalis. PA: pulmonary artery; RPA: right pulmonary artery; LPA: left pulmonary artery; AO: aorta; RV: right ventricle; RA: right atrium; LV: left ventricle; LA: left atrium; ASD: atrial septal defect; FO: fosa ovalis.

The diagnosis of inferior STEMI proved to be incorrect, and, consequently, coronarography was not performed.

The patient had a simplified Pulmonary Embolism Severity Index (sPESI) of 2, which indicated a 30-day mortality risk of 10.9% [1]. On the other hand, our patient had a high risk of bleeding (moderate anemia, on-going mild metrorrhagia and loading doses of aspirin and clopidogrel). Although pharmacologic thrombolysis imposed a double risk of systemic embolization and bleeding, the patient had severe signs of low cardiac output and severe RV dysfunction. We therefore decided in favor of systemic thrombolysis with alteplase, together with a continuous infusion of unfractionated heparin, surgical embolectomy being unavailable on site and the condition of the patient life-threatening.

There was an initial progressive improvement in hemodynamic parameters following thrombolysis. BP raised at 100/60mmHg, with a low dose of vasopressor support, oxygen saturation increasing to 98%. A new TTE showed no intracardiac thrombus (Supplementary material 5). One hour after the end of thrombolysis, she developed severe abdominal pain, anuria, and hematuria, associated with a drop in the hemoglobin level with 2g/dl. An emergency abdominal CT-scan showed complete occlusion of both renal arteries ( Figure 3 , Panel A and B), without signs of hemorrhagic complications. A multi-disciplinary team, consisting of cardiologists, radiologists and critical care physicians decided to apply the interventional aspiration thrombectomy in order to save renal function, despite high hemorrhagic risk, with a clear imagistic improvement of the renal flow but without clinical and biological improvement ( Figure 3 Panel C, D, E, and F).

**Figure 3:** Abdominal Computed Tomography after thrombolysis showing occlusion of both renal arteries. Panel A - transversal section – renal arteries are seen only at the emergence, followed by complete occlusion of both arteries. Panel B - 3D reconstruction, showing no blood flow in the renal arteries. Panel C and D: the absence of flow/low flow in right and left renal arteries, before thrombectomy. Panel E and F: flow in both renal arteries after successful aspiration thrombectomy.

After the interventional procedure, she was transferred to the intensive care unit for advanced support and dialysis. Unfractionated heparin was continued. However, the patient rapidly developed severe multi-organ system failure and died 24 hours after admission.

## Discussion

High-risk APE, with large hyper-mobile thrombus entrapped in an interatrial communication is very rare and involves high mortality and morbidity, including an increased likelihood of paradoxical embolism.^2^

In our case, there was an “in-transit” thrombus^3^ that extended from the RA, both through an interatrial communication to the LA and through the mitral valve to the left ventricle, as well as through the tricuspid valve to the right ventricle.

Optimal management of such a clinical scenario is much debated. Management strategies include thrombolysis, anticoagulation, and/or surgical extraction, with no consensus over the superiority of one.^4^ Systemic thrombolytic therapy is the recommended attitude for high-risk PE (class I, level B).^1^ As an alternative, surgical pulmonary embolectomy (class I, level C) should be considered for patients with high-risk PE, in whom thrombolysis is contraindicated.^1^ Recent experience supports combining extracorporeal membrane oxygenation (ECMO) with surgical embolectomy, in patients with high-risk PE with or without cardiopulmonary resuscitation.^1^ Surgical embolectomy has shown a trend toward improved survival, but the postoperative mortality rate is high.^5^ Randomized trials regarding management options (surgical or medical) are not feasible due to the rarity of the condition, and the literature builds on case reports.

Thrombolysis adds the risk of thrombus fragmentation and distal embolization. A report by Chartier et al. showed that there was no significant difference between anticoagulation with heparin, thrombolysis, or surgical removal in terms of in-hospital mortality.^6^ Torbicki et al.^4^ stated that the favorable result after thrombolysis could be related to the greater availability and the shorter period from diagnosis to treatment. Ferrari et al.^7^ showed that after thrombolysis, 50% of the clots disappeared within 2 hours, and the remainder within 12–24 hrs. Some studies state that in the case of high-risk APE with intracardiac thrombus, the best therapeutic attitude is surgical thrombectomy, especially in the case of patients with interatrial communication.^8^ However, surgical thrombectomy is not readily available in many centers and carries the risk of delay for treatment, general anesthesia, and cardiopulmonary bypass. In a systematic review, surgical thrombectomy was associated with a 30-day mortality of 10.8%, significantly lower when compared to thrombolysis (26.3%) and anticoagulation (25.6%).^9^

Guidelines recommend the use of catheter-directed lytics in intermediate-high-risk PE with relative contraindications to thrombolysis and use of catheter-directed thrombectomy in patients with absolute contraindications to thrombolytics or failed thrombolytic therapy. Among the percutaneous approaches that have been used alone or in combination in patients with an absolute contraindication to thrombolysis are: thrombus fragmentation aspiration thrombectomy, rheolytic thrombectomy, suction embolectomy, and echocardiogram-assisted thrombolysis.^10^

However, thrombus fragmentation techniques imply a significant risk of distal and proximal embolization. Rheolytic thrombectomy catheters function by creating a vacuum behind an area of high-pressure saline jets at the tip of the catheter, which enables simultaneous thrombus fragmentation and aspiration. Suction embolectomy is capable of achieving thrombus removal without the side effects associated with fragmentation and rheolytic techniques.^10^ Ultrasound-assisted catheter directed thrombolysis, which combines emission of low energy ultrasound and infusion of a thrombolytic agent via a multi side-hole containing catheter, is superior to heparin anticoagulation alone in improving right ventricular dilatation within 24 hours, without major bleeding complications, but it is still rarely available.^10^

As suggested in current guidelines, these treatment strategies might have been of great value in our case. Still, there are no published head-to-head trials comparing the various systems or comparing catheter-directed therapy to systemic thrombolysis. Therefore, the choice of modality should be based on local expertise and availability. None of the above-mentioned treatment strategies were available in our hospital, and the patient was extremely unstable to be transported to other hospitals. Moreover, right-to-left cardiac shunt and concomitant left side thrombosis, as in our case, are absolute contraindications to Catheter-Directed Thrombolysis.^10^

The present case opted in favor of systemic thrombolysis, which was readily available, because the patient had severe RV dysfunction and severe low cardiac output.

More recently, in the context of the Covid-19 pandemic, there have been reported cases of APE and thrombus-in-transit straddling a patent foramen ovale, 28 days after COVID symptom onset.^10^ Pro-inflammatory and pro-thrombotic states explain the hypercoagulability associated with SARS-CoV-2 infection.^11^ It is not yet well known how long this hypercoagulable state persists after recovery from infection.^11^ More data are needed to determine the optimal duration of anticoagulation.^11^ There are no data regarding surgical thrombectomy in this context.

## Conclusions

This study therefore presents a very rare case of high-risk pulmonary embolism, with a large hyper-mobile thrombus entrapped in an interatrial septum defect, crossing the mitral and aortic valves, also carrying a high-risk of systemic embolism. Our patient experienced an even more rare fatal embolic complication after applying systemic thrombolysis, a bilateral renal artery occlusion. This case illustrates the difficult decisions that clinicians may come across in everyday practice. It was necessary to balance the pros and cons of pharmacological thrombolysis, as surgical embolectomy was not available, and the evidence of the superiority of the surgical embolectomy in hemodynamically unstable patients with severe RV dysfunction is still controvertial. The use of the multi-modality imaging in all steps of treatment decisions is also emphasized.

## * Supplemental Materials

For additional information, please click here.

See the Video1, please click here.

See the Supplemental Material 3, please click here.

See the Supplemental Material 4, please click here.

See the Supplemental Material 5, please click here.
